# Influence of the duration of breastfeeding on quality of muscle function during mastication in preschoolers: a cohort study

**DOI:** 10.1186/1471-2458-12-934

**Published:** 2012-10-31

**Authors:** Simone Capsi Pires, Elsa Regina Justo Giugliani, Fernanda Caramez da Silva

**Affiliations:** 1 Postgraduate Program in Medical Sciences: Child and Adolescent Health, Universidade Federal do Rio Grande do Sul (UFRGS), Ramiro Barcelos 2400, CEP 90035-003, Porto Alegre, RS, Brazil; 2 School of Medicine, UFRGS, Ramiro Barcelos 2400, CEP 90035-003, Porto Alegre, RS, Brazil

## Abstract

**Background:**

There is some evidence of the benefits of breastfeeding to masticatory function, but no studies have evaluated the influence of breastfeeding duration on the quality of this function. The objective of this study was to investigate the association between duration of breastfeeding and quality of masticatory function in preschoolers.

**Methods:**

Cross-sectional study nested in a contemporary cohort of 144 randomly selected Brazilian infants. Data on sociodemographic, dietary, and sucking-related parameters were collected shortly after birth and at 7, 30, 60, 120, and 180 days of life. Masticatory function was assessed between the ages of 3 and 5 years, using a standardized procedure involving three foodstuffs of different consistencies, for evaluation of incision, lip competence, masticatory patterns, masticatory movements, and perioral muscle use. The quality of masticatory function was scored, and multiple linear regression was used to test for association between this score and the duration of breastfeeding.

**Results:**

A positive correlation was found between duration of breastfeeding and masticatory function scores (*r*_s_ = 0.473; p < 0.001). Children breastfed for at least 12 months had significantly higher average scores, regardless of bottle-feeding or pacifier use. Children who were breastfed for longer were more likely to score satisfactorily across all tested parameters.

**Conclusions:**

Breastfeeding has a positive impact on mastication. In our sample, duration of breastfeeding was positively associated with the quality of masticatory function at preschool age.

## Background

Many child health benefits have been attributed to breastfeeding, including the promotion of adequate oral motor development, which has a positive effect on growth and craniofacial development [1-4]. During breastfeeding, intense movement of the lips, tongue, mandible, maxilla, and cheeks occurs, with beneficial effects on the infant’s oral motor development. The jaw movements involved in extraction of milk from the breast provide major stimuli for growth of the temporomandibular joint and, consequently, encourage harmonious growth and development of the facial region [2]. The muscles involved in breastfeeding, particularly the masseter, are the same muscles that will later (from the age of 6 months onward) carry out mastication [5,6]. Therefore, mastication continues the process of stimulation of the orofacial muscles that began with sucking at the breast. When performed correctly, it also plays a role in the development of the maxilla and mandible and contributed, together with genetic and environmental factors, to the stability of dental occlusion and functional and muscle balance. Whereas sucking at the breast has a favorable effect on masticatory function [5,7-9], other forms of sucking, such as those involved in bottle-feeding and pacifier use, produce different functional stimuli, which may jeopardize oral motor development and the position and strength of stomatognathic structures, with a detrimental impact on oral functions, including mastication [1,6,9,10].

If, on the one hand, there is evidence of the benefits of breastfeeding to masticatory function and of the negative impact of bottle-feeding and pacifier use on development of the stomatognathic system [1-3,5,6,9-11], no studies have evaluated the influence of breastfeeding duration on the quality of masticatory function. The present study was designed to bridge this gap. We investigated the association between duration of breastfeeding and quality of masticatory function in preschoolers with complete deciduous dentition, to test the hypothesis that, the longer the duration of breastfeeding, the better the quality of masticatory function.

## Methods

This was a cross-sectional study nested within a contemporary cohort of children followed from birth to the ages of between 3 and 5 years. Children were selected from the rooming-in facilities of Hospital de Clínicas de Porto Alegre, a general, public, university-affiliated, Baby-Friendly Hospital in the city of Porto Alegre, Brazil, where approximately 3500 children are delivered each year.

Sampling consisted of random daily selection of two healthy neonates who met the following eligibility criteria: had a birth weight ≥2500 g, had begun breastfeeding, and had families living in the municipality of Porto Alegre, state of Rio Grande do Sul, Brazil. Twins were excluded from the study, as were infants who could not be kept in a rooming-in arrangement due to issues involving themselves or their mothers.

Sociodemographic data were collected by means of interviews of the participating infants’ mothers, conducted at the maternity ward between the second and third day after delivery. Data on dietary and sucking habits were collected at 7 and 30 days through home visits and at 60, 120, and 180 days through telephone interviews, or, when telephone contact could not be obtained, through home visits. When participants were aged between 3 and 5 years, their parents or guardians were contacted by phone and asked to take their children for assessment of masticatory function. When families could not be reached by phone, home visits were scheduled.

Masticatory function assessment was performed by a speech therapist in a private office setting. Assessment was conducted at home if children failed to report for in-office examination as scheduled. The examiner was blinded to dietary and non-nutritive sucking habits. Only after examination had been completed was the mother or guardian asked to provide information on the infant’s diet and sucking habits, from the age of 6 months through the date of assessment.

The investigator began by explaining the examination procedures to the child, using age-appropriate language.

Three foodstuffs of different consistencies, requiring distinct muscle actions for mastication, and commonly well-accepted by children were chosen for assessment: banana (Prata), Brazilian cheese bread (cold), and breadsticks. At least 60 seconds were allowed to elapse between assessments with each food.

Five parameters of masticatory function were assessed: incision, lip competence, masticatory pattern, masticatory movements, and use of the perioral muscles. These parameters were considered satisfactory when the following criteria were met: incision – use of the incisors for cutting the provided food [12,13]; lip competence – mastication performed predominantly with the lips sealed [14]; masticatory pattern – bilateral, alternating masticatory movements [14]; masticatory movements – predominantly rotational mandibular motion [15-17]; perioral muscle use – no excessive utilization of the perioral muscles during mastication [18,19]. The quality of masticatory function was scored on a simple scale based on the sum of points. All masticatory functional parameters were dichotomously scaled as satisfactory (1 point) or non-satisfactory (0 point) according to the child’s performance. As five parameters were assessed during mastication of three different foods, total scores could range from zero (poorest masticatory performance) to 15 (best masticatory performance).

All assessments were filmed, using a model SV-AV 25 D-snap digital camcorder (Panasonic, Osaka, Japan), for later observation of posture and masticatory movements. Footage of each participant was assessed separately by the study investigator and by an independent speech therapist. If there was no consensus between assessments, a third independent speech therapist was asked to provide an opinion, and the result with the most agreement was accepted. Assessment was based on mastication of the second bite of each tested food whenever possible, but, in some cases, due to the age of the subjects and their difficulty with the task, assessment was based on the morsel of food for which mastication was most visible. If the child accepted only one piece of the provided food, assessment was based on this sole instance of mastication.

Data were processed in Microsoft Excel 2003 (Microsoft Corporation, Redmond, WA, USA). The double data entry method was used. Statistical analyses were conducted in the Statistical Package for the Social Sciences 13.0 (SPSS Inc., Chicago, IL, USA) software environment.

Continuous variables were expressed as means and standard deviations (when symmetrically distributed) or medians and ranges (when asymmetrically distributed), and categorical variables, as absolute and relative frequencies. The following statistical tests were employed: Student’s *t* test, for comparison of mean mastication scores; Pearson’s chi-square test, for comparison of frequencies; Spearman’s correlation coefficient, to test for correlation between mastication scores and duration of breastfeeding; and multiple linear regression, to test for association between breastfeeding and quality of masticatory function. In the logistic regression model, we included variables often associated with masticatory performance (bottle-feeding, pacifier use, and thumb sucking) that exhibited p < 0.20 on bivariate analysis, in addition to sociodemographic variables such as age and gender of the child and age, educational attainment, and parity of the mother. The significance level was set at 5% (p ≤ 0.05).

The present study was approved by the Health Research and Ethics Committee of the Hospital de Clínicas de Porto Alegre Graduate Research Group. Written informed consent was provided by the mothers or legal guardians of all participating subjects. After assessment, the mothers or legal guardians of all children who used pacifiers and/or were bottle-fed were informed of the negative consequences of these habits on orofacial development and of the benefits of providing foods that encourage masticatory muscle activity for proper growth and development of the anatomical structures involved in mastication.

## Results

**Figure 1:**
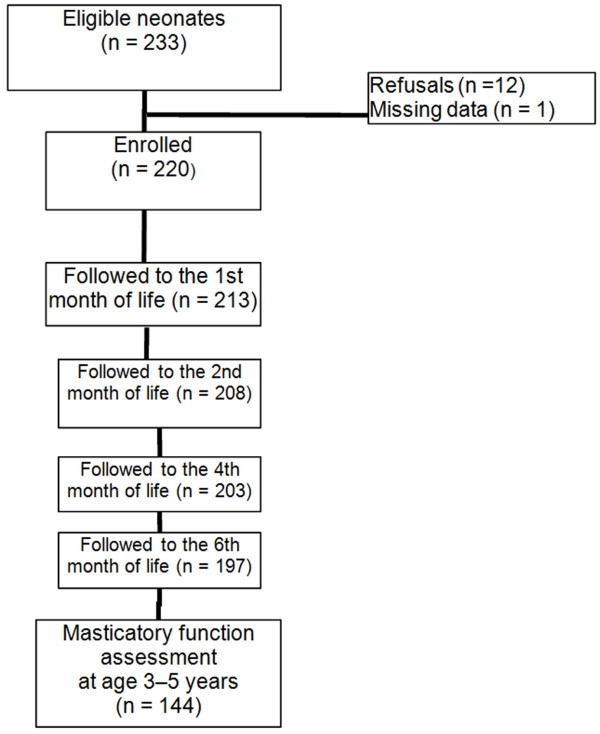
Cohort follow-up flowchart.

Assessment of masticatory function was performed in 144 of the 220 children originally included in the cohort. A total of 76 children were lost to follow-up: 61 due to failure to locate the participant’s family, nine due to refusal to eat the provided foods, three due to refusal to remain in the study, and three who had moved away (Figure 1).

The overall profile of the children lost to follow-up was similar to that of the final study sample, except with regard to skin color; there were more children born to white mothers in the loss to follow-up group than in the study completion group (82.6% vs. 63.2%). However, skin color was not associated with the quality of masticatory function (p = 0.289).

Mean age at assessment was 50.3±7.2 months (range, 40 to 64 months). Other data concerning the main characteristics of the participants, their dietary and sucking habits, and their parents are shown in Table 1. The number of children breastfed according to World Health Organization recommendations [20] was relatively small: only nine children (6.3%) had been exclusively breastfed during the first 6 months of life, and only 44 (30.6%) had been breastfed for 2 years or longer. The mean duration of breastfeeding was 10 months (95% confidence interval [95%CI], 6.8 to 13.2), and six children (4.2%) were still breastfeeding at the time of assessment. The prevalence of bottle-feeding and pacifier use at some point between birth and assessment was exceedingly high (94.4% and 76.4% respectively). Seven children (4.9%) were still bottle-fed and 30 (20.8%) still used pacifiers at the time of assessment.

**Table 1:** General sample characteristics and data on dietary and sucking habits of preschoolers, Porto Alegre, Brazil (n = 144)

**Characteristic**	**n**	**%**
Male gender	76	52.8
Firstborn	73	50.7
Maternal age ≥ 20 years at time of birth	104	72.2
Maternal educational attainment ≥ 8 years formal schooling	92	63.9
Exclusive breastfeeding, ≥ 6 months	9	6.3
Breastfeeding, ≥ 12 months	66	45.8
Breastfeeding, ≥ 24 months	44	30.6
Pacifier use, any duration	110	76.4
Pacifier use, ≥ 6 months	77	53.5
Bottle-feeding, any duration	136	94.4
Bottle-feeding, ≥ 12 months	122	84.7
Thumb sucking, any duration	14	9.7

**Figure 2:**
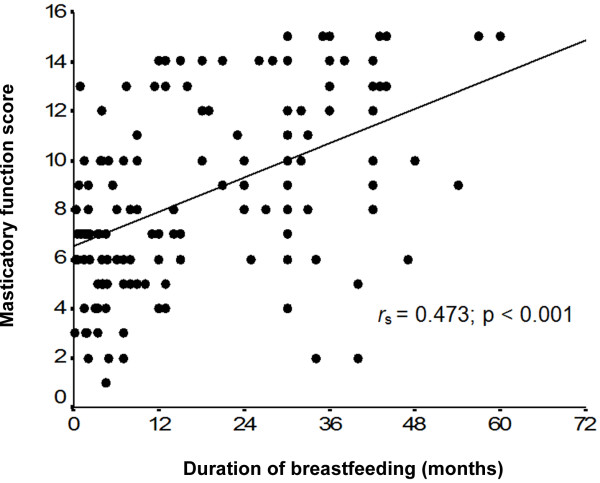
**Scatterplot of the correlation between duration of breastfeeding and masticatory function scores *r ***_**s**_**= Spearman’s rank correlation coefficient (nonparametric measure).**

The mean masticatory function score was 8.4±3.6 points (range, 1 to 15). A positive correlation was found between duration of breastfeeding and masticatory function scores (Figure 2).

Table 2 shows the mean scores obtained during assessment of mastication according to variables often associated with masticatory performance. Mean masticatory function scores were significantly higher among children who had been breastfed for 12 months or longer, had been bottle-fed for no longer than 12 months, and had not used pacifiers for longer than 6 months. Thumb sucking was not significantly associated with masticatory function scores on bivariate analysis (p = 0.602), and did not reach the level of significance required for inclusion in the linear regression model (p < 0.20).

**Table 2:** Masticatory function scores in preschoolers, according to type of feeding and sucking habits, Porto Alegre, Brazil (n = 144)

**Variable**	**Masticatory function score**		
	**n**	**Mean ± SD**	p*
Breastfeeding (months)			
< 12	78	6.79±2.84	<0.001
≥12	66	10.4±3.53	
Bottle-feeding (months)			
< 12	22	12.4±2.67	<0.001
≥ 12	122	7.73±3.33	
Pacifier use (months)			
< 6	67	10.1±3.48	<0.001
≥ 6	77	7.00±3.15	
Thumb sucking			
Yes	14	8.92±3.83	0.602
No	130	8.39±3.63	

SD, standard deviation.

*Student’s *t* test.

The results of the multivariate analysis are shown in Table 3. We detected a significant association between maintenance of breastfeeding for 12 months or longer and superior masticatory function. On average, children who had breastfed for 12 months or longer scored two points higher on our masticatory function scale, regardless of bottle-feeding or pacifier use. Conversely, use of these devices was associated with a 3-point or 1.4-point reduction in masticatory function scores respectively.

**Table 3:** Variables associated with masticatory function scores*, Porto Alegre, Brazil (n=144)

**Variable**	**Score**	
	**Slope (95%CI)**	**p**
Breastfeeding, ≥ 12 months	2.0 (0.7 to 3.2)	0.001
Bottle-feeding, ≥ 12 months	−3.2 (−4.8 to −1.7)	< 0.001
Pacifier use, ≥ 6 months	−1.4 (−2.5 to −0.2)	0.016

95%CI, 95% confidence interval.

Coefficient of determination (*R*^2^) = 35.9%; F_(3,140)_ = 26.2; p < 0.001.

*Adjusted for age and gender of the child and for age, educational attainment, and parity of the mother.

Table 4 shows the results of masticatory function assessment, stratified by each of the five tested parameters, according to the duration of breastfeeding. Prevalence ratios represent the odds that children breastfed for 12 months or longer would score satisfactorily on each parameter as compared with children who were breastfed for shorter periods. Children who had breastfed for longer had higher odds of satisfactory scores across all five parameters and all three different foods tested, particularly in the masticatory pattern and masticatory movement parameters.

**Table 4:** Number of children with satisfactory masticatory functions according to duration of breastfeeding

**Variable**	**BF [n (%)]**		**PR(95%CI)**
	**< 12 mo(n = 78)**	**≥ 12 mo(n = 66)**	
Incision	26 (33.3)	37 (56.1)	1.68 (1.15 to 2.46)
Lip competence	44 (56.4)	49 (74.2)	1.32 (1.03 to 1.68)
Masticatory patterns	2 (2.6)	14 (21.2)	8.27 (1.95 to 35.1)
Masticatory movements	6 (7.7)	31 (47.0)	6.11 (2.72 to 13.7)
Perioral muscle use	29 (37.2)	45 (68.2)	1.83 (1.32 to 2.56)

BF, breastfeeding; 95%CI, confidence interval; PR, prevalence ratio.

## Discussion

Although there is substantial evidence that breastfeeding has a positive impact on masticatory function [5-7], this was the first study to show a positive association between the duration of breastfeeding and the quality of masticatory function in preschoolers with complete deciduous dentition. This association may be explained, at least in part, by the purported role of breastfeeding as a promoter of healthy development of the muscles later responsible for masticatory function, particularly the masseter muscles [5,6,21-23]. The cycle of movements performed by the child’s jaws while sucking at the breast enables adequate growth and positioning of the jaws for proper tooth eruption [5-24], which, in turn, plays an essential role in learning to chew properly. Furthermore, children who breastfeed are less likely to be exposed to other forms of sucking, such as bottle-feeding and pacifier use [25], which are known to be deleterious to the development of the oral cavity [1,9,10].

Masticatory function assessment revealed that children breastfed for 12 months or longer scored higher across all tested parameters, but particularly on the masticatory pattern and masticatory movement items, which are those most strongly influenced by breastfeeding due to their close association with development of the muscles of mastication. Nevertheless, a high proportion of children who had been breastfed for at least 1 year scored poorly on the various tested parameters. This finding was probably associated with the high prevalence of bottle-feeding and pacifier use in the study population (94.4% and 76.4% respectively), even among breastfed children. Several studies have shown the negative influence of both devices on masticatory function [6,9,26]. Our study confirmed that bottle-feeding and pacifier use have a negative effect on children’s oral motor function, since children who were bottle-fed for longer than 1 year and those who used a pacifier for longer than 6 months scored lower on masticatory function assessments. A potential explanation for this finding is that sucking at a pacifier or bottle teat primarily involves the perioral muscles, which provide no stimulus to the temporomandibular joint and, therefore, do not encourage mandibular growth [1-5]. It is important to emphasize that, according to the results of the logistic regression analysis in the present study, the effects of breastfeeding and bottle-feeding or pacifier use on masticatory function were independent, i.e., even if children use bottles and/or pacifiers, breastfeeding still have a positive impact on mastication. This means that the masticatory function of children who were bottle-fed and/or used pacifier in this study would probably be more strongly affected without the mastication-stimulating muscle movements provided by the suction on the breast.

During the course of this study, some methodological approaches were used to minimize the possibility of bias. Follow-up during the first 6 months reduced the risk of recall bias with respect to certain exposure factors; the choice of three different foodstuffs with different textures and high acceptance by children made assessment of masticatory function more realistic in terms of participant exposure to the tested factors; independent evaluation of assessment footage by at least two trained professionals reduced the risk of measurement bias; and the use of a multivariate analysis model helped control for potential confounding between factors believed to be involved in masticatory function.

Some potential limitations of this study should be mentioned. Active search of all participants failed to prevent substantial loss to follow-up. Such losses are common in studies of this type and design, particularly when samples are composed of the children of young adults living in the peripheral areas of large cities in a developing country. However, the similar profiles of the group lost to follow-up and of the final study sample suggest that selection bias is unlikely. The possibility of memory bias should be considered, since there was a long gap in follow-up after the initial 6 months. However, mothers tend to recall the date they stopped breastfeeding with relative precision. A U.S. study found no significant differences in BF duration reported after 6 months and at 1 to 3.5 years [27]. Furthermore, the fact that the outcome was not based on a specific date, but on a period (12 months or more), undoubtedly reduces any memory bias. The age range at assessment of masticatory function (3 to 5 years) might be a concern, since we cannot disregard the possibility of some improvement of oral function with age. Nevertheless, mean masticatory function scores were no different for children aged 3, 4, or 5 years in our study (8.3, 8.6 and 8.6 respectively).

Even though exclusive breastfeeding until the age of 6 months is known to provide stimuli essential to orofacial growth and development [1,2], only nine children in our sample had been exclusively breastfed for this duration, which precluded assessment of the potential association between exclusive breastfeeding and masticatory function. If our sample had consisted of (a) a greater number of children who had been breastfed for at least 1 year (with at least 6 months of exclusive breastfeeding), did not bottle-feed and did not use pacifiers, and (b) an equal number of children who had been exclusively bottle-fed, our findings would probably have been far more striking.

## Conclusions

Breastfeeding seems to have a positive impact on mastication, as duration of breastfeeding was positively associated with the quality of masticatory function in preschool age, regardless of the use of bottles and/or pacifier. In light of the short duration of breastfeeding in Brazil [28], this finding further stresses the importance of actions designed to promote, protect, and support breastfeeding. In addition to its well-established benefits, breastfeeding appears to have a positive effect on masticatory function, ensuring functional stimuli for proper facial growth and development.

## Competing interests

The author(s) declare that they have no competing interests.

## Authors' contributions

SCP: Conception and design of the study; acquisition, analysis and interpretation of data; drafting the article. EJG: Conception and design of the study; analysis and interpretation of data; drafting the article. FCS: Conception and design of the study; acquisition, analysis and interpretation of data; drafting the article. All authors read and approved the final manuscript.

## Pre-publication history

The pre-publication history for this paper can be accessed here:

http://www.biomedcentral.com/1471-2458/12/934/prepub
